# A Computational Model of Deep-Brain Stimulation for Acquired Dystonia in Children

**DOI:** 10.3389/fncom.2018.00077

**Published:** 2018-09-20

**Authors:** Terence D. Sanger

**Affiliations:** Department of Biomedical Engineering, Biokinesiology, and Child Neurology, University of Southern California, Los Angeles, CA, United States

**Keywords:** deep brain stimulation, pediatric, dystonia, basal ganglia, thalamus, single unit recording

## Abstract

The mechanism by which deep brain stimulation (DBS) improves dystonia is not understood, partly heterogeneity of the underlying disorders leads to differing effects of stimulation in different locations. Similarity between the effects of DBS and the effects of lesions has led to biophysical models of blockade or reduced transmission of involuntary activity in individual cells in the pathways responsible for dystonia. Here, we expand these theories by modeling the effect of DBS on populations of neurons. We emphasize the important observation that the DBS signal itself causes surprisingly few side effects and does not normally appear in the electromyographic signal. We hypothesize that, at the population level, massively synchronous rhythmic firing caused by DBS is only poorly transmitted through downstream populations. However, the high frequency of stimulation overwhelms incoming dystonic activity, thereby substituting an ineffectively transmitted exogenous signal for the endogenous abnormal signal. Changes in sensitivity can occur not only at the site of stimulation, but also at downstream sites due to synaptic and homeostatic plasticity mechanisms. The mechanism is predicted to depend strongly on the stimulation frequency. We provide preliminary data from simultaneous multichannel recordings in basal ganglia and thalamus in children with secondary dystonia. We also provide illustrative simulations of the effect of stimulation frequency on the transmission of the DBS pulses through sequential populations of neurons in the dystonia pathway. Our experimental results and model provide a new hypothesis and computational framework consistent with the clinical features of DBS in childhood acquired dystonia.

## Introduction

Deep brain stimulation (DBS) is a neuromodulatory intervention that has had profound impact on several adult-onset movement disorders, including parkinsonism, tremor, and dystonia (Montgomery, 2010). In children, DBS is most often used to treat dystonia, with significant benefit in up to 90% of children with primary or genetic dystonias (Vidailhet et al., 2005). Unfortunately, in the much larger group of children with secondary dystonia due to acquired brain injury, only 50% of children have significant benefit from DBS (Vidailhet et al., 2009). While this may reflect the irreversibility of destructive neural lesions in acquired forms of dystonia, the gradual onset of dystonia following acute injury suggests that the movement disorder is more likely an emergent property of disordered neural computation rather than a direct consequence of the injured region (Peterson et al., 2010). Therefore, there is reason to hope that modulation of neural activity could slow, prevent, or reverse dystonic symptoms, even when the underlying injury cannot be repaired.

Evidence for at least partial reversibility of childhood acquired dystonias comes from studies of medications. For example, high-dose anticholinergic medication can ameliorate symptoms, particularly in younger children or those with less severe symptoms at the time of initiation of treatment (Sanger et al., 2007). While the dramatic effects of dopaminergic medications in primary dopamine-responsive dystonias are not replicated in acquired dystonias, some degree of improvement is often seen, even when symptoms have been longstanding at the time of initiation of treatment (Bastian, 2000). Therefore, in medication-refractory cases there is reason to hope that DBS could be effective.

Unlike medications, DBS can be targeted at specific brain regions and therefore provides anatomic specificity. However, DBS remains a very crude intervention compared to the exquisite control of neural activity that would normally be present. It is therefore, “neuromodulation” (Oshima et al., 2015; Ashkan et al., 2017); the most we can hope for is to up-regulate or down-regulate the activity or change excitability thresholds of target regions. DBS does not inject normal patterns nor can it selectively modify abnormal patterns. Nevertheless, it can have significant benefits on motor function (Wichmann and DeLong, 2016). Of course, brain injury can also be considered more modulatory than selective. Most childhood acquired brain injury affects multiple cell types over a large volume of brain, so that the clinical effect is most likely due to scaling of input to downstream targets and lack of transmission of information, rather than a deficit of specific patterns of information. Therefore, DBS as an intervention may be well-matched to the functional consequences of brain injury.

Just as medications can have very different mechanisms of action in different disorders, it is likely that DBS has very different mechanisms of action that depend on the underlying disorder, the targeted brain region, and the stimulation parameters (Udupa and Chen, 2015). For example, stimulation of subthalamic nucleus (STN) or internal globus pallidus (GPi) in Parkinson's disease can have immediate effects on bradykinesia, whereas stimulation of GPi in either genetic or acquired dystonias usually has effects only weeks or months later (Herrington et al., 2016).

Here, we will focus on the use of DBS to treat acquired dystonias in children (Albanese et al., 2013). The most common cause of acquired dystonia in childhood is cerebral palsy (CP), with a common mechanism being acute hypoxic-ischemic injury at term birth (Sanger, 2003). This mechanism is known to cause injury to both the medial pallidum (including GPi) and the lateral thalamus (including the GPi projection areas) (Volpe et al., 2017). In more severe cases, injury to other motor areas (including motor cortex in precentral gyrus and cranial nerve nuclei) and sensory areas (including sensory cortex in postcentral gyrus and ascending sensory pathways in the posterior limb of the internal capsule) likely contributes to symptoms. Other causes include inflammatory, infectious, neurometabolic, and toxic injuries that often have a predilection for pallidum, striatum, and/or thalamus (Sanger, 2005). In some cases the injury is transient or reversible, but in most cases it is either static or, in the case of neurometabolic and some genetic disorders, progressive.

The purpose of our model is to provide a summary of current knowledge related to the mechanism of DBS for this class of disorders. With existing technology there are very limited options in terms of parameters for DBS, and targeting is limited by the use of standardized anatomic atlases and lack of ability to understand the information processing deficit responsible for symptoms in each child. Our goal is that this model will facilitate clinical interventions, clinical research, and device development in order to make DBS more effective for a wider group of children.

## Background

### Current models of basal ganglia function

Despite many years of research, the role of the basal ganglia in dystonia remains mysterious. Much of clinical care has been guided by the “rate model” (Vitek and Giroux, 2000). This model is based on the pattern of excitatory and inhibitory connections between cortex, basal ganglia, and thalamus, and it posits that parkinsonism and dystonia are associated with very high and very low activity in GPi, respectively. In particular, because the output of GPi is carried by inhibitory synapses, and because the output of thalamus is often excitatory to cortex, the rate model suggests that high GPi activity in parkinsonism leads to decreased cortical activity, whereas low GPi activity in dystonia leads to increased cortical activity.

While this model has had strong influence on clinical practice, it is inconsistent with newer evidence from neuroanatomy, clinical results, and electrophysiology in humans and nonhuman primates (Wichmann and DeLong, 1996). For example, while recordings from GPi in primary dystonia do show decreased rates from what is seen in parkinsonism, lesions in GPi that would further decrease inhibition on thalamus are known to ameliorate genetic dystonia. Our recording data show that during dystonic spasms, activity in both GPi and its projection regions in thalamus (VA and Voa/Vop nuclei) increase together. This is not predicted by the rate model, because GPi would be expected to decrease during spasms, and GPi and thalamic projection areas should anti-correlate. Furthermore, since the output of GPi is inhibitory, there must be some excitatory input to thalamus. The most likely candidate is corticothalamic projections (Rovo et al., 2012), although an alternative possibility is that the thalamic response to input from GPi includes a rapid rebound excitation so that the net effect of GPi on thalamus might be excitatory (Rovo et al., 2012; Herrington et al., 2016).

### Current models of deep brain stimulation mechanism

Clinical practice has been based on the observation that DBS has similar effects to lesions in GPi, subthalamic nucleus (STN), and motor thalamus (Anderson et al., 2003; McIntyre et al., 2004b). The inhibition was originally explained as “depolarization block” meaning that neurons near the stimulating electrode are depolarized so frequently that they are unable to transmit other information. If high-frequency stimulation (above 120 Hz) causes depolarization block, then the possibility exists that low-frequency stimulation (below 60 Hz) might depolarize downstream regions. However, attempts to use low-frequency stimulation as the opposite of high-frequency stimulation have not been as successful as predicted. In dystonia, both low and high-frequency stimulation have been shown to be effective, although not always in the same patient (Herrington et al., 2016; Ashkan et al., 2017). Simulation results suggest that DBS may be inhibitory at cell bodies or dendrites while simultaneously causing axonal depolarization and therefore propagation of the stimulus (McIntyre et al., 2004a; Birdno et al., 2014). These models address the effect of DBS on single neurons in GPi and thalamus, extrapolating the aggregate population response from the predicted response in individual cells.

### Basis for the model

In order to understand the mechanism of DBS, we propose to look directly at its effect on populations of neurons, and how the population activity is transmitted to populations in other regions downstream from the stimulation. For example, DBS in GPi causes changes in the firing of GPi neurons, but it also will cause changes in thalamic and eventually motor cortical neurons. DBS in thalamus will cause changes in motor cortical neurons. And because there are multiple connections between thalamus and basal ganglia and reciprocal connections between cortex and thalamus, there may be complex re-entrant effects of the stimulation. The effects of DBS will have both immediate components in terms of blockade of transmission or the neural effects of the stimulation pulses, as well as delayed effects due to compensatory plasticity. Plasticity will be mediated by synaptic mechanisms, but could also be mediated by homeostatic plasticity mechanisms (Keck et al., 2017). The role of plasticity seems to be particularly important for stimulation in GPi, in which the effects can be delayed by weeks or months following changes in stimulation (Ashkan et al., 2017).

### Effects of stimulation frequency

Based on clinical observations, we propose that at the highest frequencies (above 200–250 Hz), the stimulated neurons may be held in permanent depolarized states and thus no firing at all would occur, with effects similar to a local lesion. At slightly lower frequencies of stimulation (~100–200 Hz), DBS may cause depolarization blockade in the sense that incoming neural signals are unable to propagate through the stimulated region because all incoming signals occur either at the time of a stimulation or during the absolute refractory period. Although DBS will block incoming neural signals, it will also intermittently depolarize the stimulated cell bodies or nearby axons leading to transmission of regular spiking to downstream recipients. In this case, DBS will substitute chronic regular output for the abnormal pattern of output that would have otherwise been transmitted. At still lower frequencies (30–50 Hz), some of the incoming activity may be propagated during the inter-stimulus interval (ISI) so that the output is a mix of the DBS pulse plus the incoming voluntary and involuntary neural activity. These frequencies are only illustrative because the actual function depends on the refractory period, the strength of the DBS, the presence of rebound firing, the relative effect of stimulation on excitatory and inhibitory neurons, and many other factors that are difficult to measure. The important consequence is that by appropriate selection of frequency it may be possible to create varying combinations of blockade of upstream information and stimulation of downstream structures. The net effect would be the addition of rhythmic downstream stimulation (for low frequencies), substitution of rhythmic downstream stimulation (for high frequencies), or complete blockade (for the highest frequencies).

### Effects on plasticity

The delayed response to GPi stimulation suggests that plasticity mechanisms may be contributing to efficacy. This could be due to long-term potentiation (LTP) at synapses immediately downstream from stimulation. Another possibility is LTP or long-term depression (LTD) at synapses that compensate for or inhibit the effects of the stimulation. Very low stimulation frequencies (below 2 Hz) may be able to cause LTD in downstream structures, further contributing to blockade of transmission. Frequencies above 10 Hz may have the ability to cause LTP in downstream structures (Bliss and Cooke, 2011).

We hypothesize that as stimulation frequency increases, the persistent depolarization at the stimulation target as well as downstream regions will lead to homeostatic plasticity in addition to Hebbian synaptic plasticity (Turrigiano, 1999; Keck et al., 2017). In particular, it is reasonable to conjecture that thresholds for activation by other inputs will increase in any area that receives the DBS input, either directly or trans-synaptically. This will decrease the response to DBS over time, but it will also decrease the response to non-DBS inputs, so it provides another possible mechanism by which DBS can have an inhibitory effect. This phenomenon has not been investigated, but homeostatic plasticity is a widely-observed phenomenon that is likely to have an effect on the response to DBS.

Neuronal injury could also have a potential effect on homeostatic plasticity (Dennis et al., 2013). Unlike primary dystonias, parkinsonism, and drug-induced dystonias, in childhood secondary dystonia there is cellular destruction that is often visible on MRI in medial pallidum and thalamus, and visible microscopically as “status marmoratus” within pallidum. In these cases, we conjecture that reduction in active cells leads to decreased thresholds downstream, so that the overall firing of target regions is maintained within effective ranges. This is seen commonly in other areas of the nervous system. For example, injury to corticospinal tract leads to changes in spinal circuits that cause excess muscle activity in the form of spasticity. Injury to anterior horn cells leads to enlarged motor units. Reduction of peripheral sensory input can lead to upregulation of primary sensory areas that is thought to be responsible for phantom limb pain, tinnitus, and allodynia (Flor et al., 2006; Møller, 2007).

We hypothesize that similar homeostatic mechanisms will increase the sensitivity of downstream target areas following upstream injury. For example, thalamic neurons would be expected to change their sensitivity to the decreased number of surviving inputs from GPi. Cortex would be expected to increase its sensitivity to the surviving inputs from thalamus, etc. The consequence of increased sensitivity is that a small number of upstream input cells will have significantly enhanced downstream effect. To the downstream structures this will appear equivalent to highly synchronized input, since each remaining upstream cell will trigger large groups of downstream cells. Synchronization is thought to be a mediator of abnormal function in primary dystonia and parkinsonism (Miocinovic et al., 2015), and thus upstream destruction could be a mechanism causing synchronization and hypersensitivity in secondary dystonia.

Therefore, neuronal injury could result in downstream threshold changes mediated by both synaptic plasticity and homeostatic plasticity. The effect of DBS could be due in part to a reversal of the abnormal plastic response to decreased neuronal input firing. For example, if upstream neuronal injury leads to abnormal reductions in downstream thresholds, then upstream DBS might lead to an increase in downstream thresholds that could reverse this effect.

### Effects of rhythmic synchronous activity

An important potential clue to the mechanism of DBS is the lack of significant clinical side effects of stimulation. Typical stimulation parameters can be above 5 V with several milliamps of current delivery. In cortex, stimulation at these levels would be expected to cause immediate contraction of muscles, and in susceptible patients seizures might be triggered (Lesser et al., 1999). Thus the intensity of the stimulation is out of proportion to the lack of side effects, suggesting that DBS does not propagate to the pyramidal output pathways of motor cortex.

DBS induces precise time-locked neural activity due to simultaneous stimulation of a large contiguous region of brain. Synchronized bursts are characteristic of sleep, and recent recording data suggests this is also the pattern present during the ictal phase of seizures (Weiss et al., 2013; Truccolo et al., 2014). In fact, precise time-locked synchrony may herald the end of a seizure (Schindler et al., 2007; Sobayo et al., 2013). Therefore, synchronized depolarizations due to DBS might appear to the brain as rest, sleep, or seizures. In contrast, desynchronized activity is common during awake behavior (Renart et al., 2010; Rothkegel et al., 2011). Voluntary movement is associated with desynchronization in motor areas (McFarland et al., 2000; Park et al., 2018), and visual perception is associated with desynchronization in occipital lobe (Tan et al., 2014). In general, desynchronized non-rhythmic activity is the hallmark of normal information processing, whereas synchronous rhythmic activity is the hallmark of inactivity, sleep, or seizures (Steriade et al., 1993).

Synchronous activity could be actively suppressed by mechanisms similar to those that are responsible for termination of seizures. Rhythmic activity may be actively suppressed by mechanisms that are responsible for temporal habituation. Therefore, we would expect that neural activity capable of generating persistent postures or movements in dystonia would be asynchronous and non-rhythmic, and DBS could function by converting this activity to synchronous rhythmic activity that is ineffective at producing muscle contraction.

### Propagation of DBS depolarization

The observation that the DBS signal does not propagate to primary output areas means that DBS can be used as neuromodulation for deep structures without generating significant sensory or motor side effects. Clinically, when side effects do occur, they are often transient suggesting that rapid plasticity mechanisms lead to habituation. This is so common that when paresthesias or muscle contractions occur in response to a change in DBS settings, patients are routinely asked to wait a few minutes “to see if it goes away,” which it usually does. Furthermore, the mechanism responsible for beneficial results is not dependent on propagation of pulses to primary sensory or motor areas.

We propose that blocking of downstream propagation of DBS not only is responsible for the relative lack of side effects, but it may also be one of the ways in which DBS can function as a lesion. The DBS signal itself is ineffective, and may lead to downstream desensitization. When DBS is performed at high frequencies, it may work by substituting an ineffective pattern for an effective but involuntary pattern.

## Model

We propose a simplified model to explain the frequency dependent response to DBS. The model considers only the trans-synaptic propagation of signals through sequential populations of neurons, and how this propagation is affected by the DBS stimulation. We examine propagation of three sources of signals. The first is the DBS stimulus itself. The second is propagation of signals from “upstream” structures that would normally innervate the region that is the target of DBS. The third is the effect on other “midstream” side inputs to downstream structures. Whenever, the DBS stimulus propagates trans-synaptically to downstream areas, it is expected to modify the responsiveness of those areas to their other inputs, perhaps by a combination of depolarization blockade, rebound excitation, or alteration of thresholds due to homeostatic plasticity.

**Figure 1:**
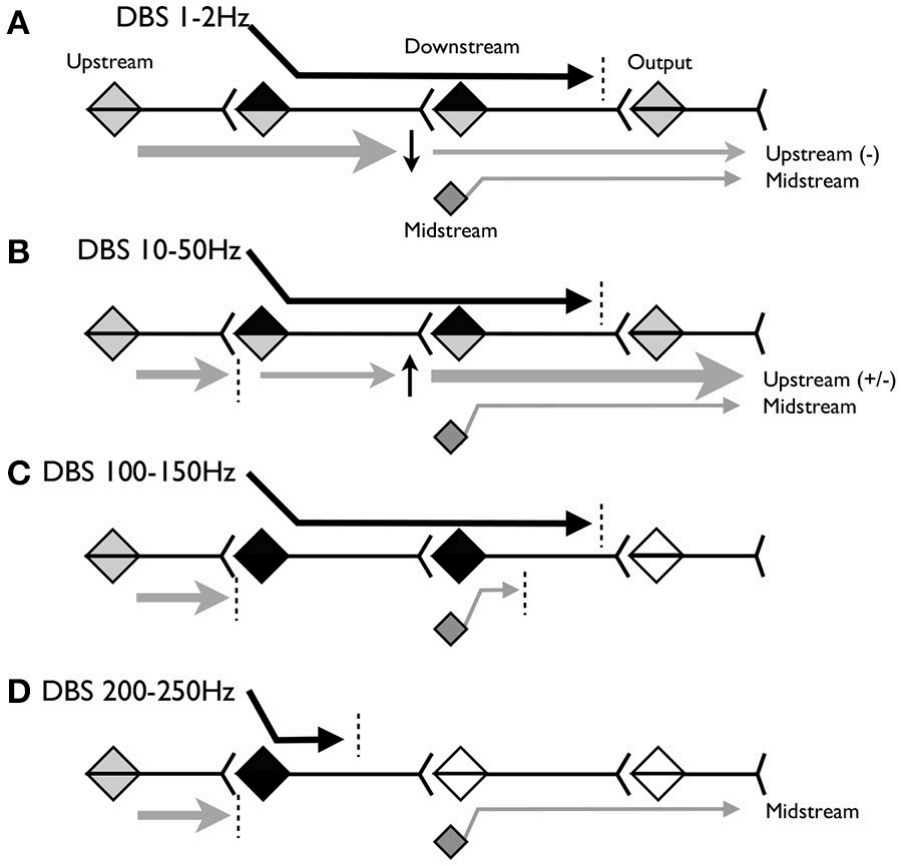
Schematic model of the hypothesized propagation of signals at various frequencies of stimulation. **(A)** Predicted effect of stimulation below 2Hz. **(B)** Predicted effect of stimulation between 10 and 50Hz. **(C)** Predicted effect of stimulation between 100 and 150 Hz. **(D)** Predicted effect of stimulation above 200 Hz.

Figure 1 illustrates the model. Figure 1A shows the predicted effect of low-frequency stimulation, below 2 Hz. At this frequency, voluntary and involuntary signals upstream of the DBS target are able to propagate during the inter-stimulus interval (ISI). Because low-frequency DBS may cause long-term depression (LTD) at the downstream target, the upstream signals may be partially attenuated but nevertheless may continue to propagate to motor outputs. Other “midstream” inputs to the downstream target may be partially blocked by depolarization and homeostatic plasticity. The DBS signal itself propagates but may be attenuated or blocked at the output synapse by mechanisms sensitive to excessive rhythmic or correlated activity.

Figure 1B shows the predicted effect of stimulation between ~10 and 50 Hz. At this frequency, the downstream synapse is expected to undergo long-term potentiation (LTP). The ISI is shorter, so less of the upstream signal will propagate, but whatever does propagate may be partially amplified due to LTP at the downstream synapse. Other inputs to the downstream target will continue to be partially blocked by depolarization block and homeostatic plasticity.

Figure 1C shows the predicted effect of stimulation between ~100 and 150 Hz. At this frequency we expect upstream signals to be completely blocked because the ISI is <10 ms and thus may be shorter than the absolute refractory period of the stimulated neurons. The DBS signal again propagates until blocked by mechanisms sensitive to correlated activity. DBS inputs to the downstream neurons will depolarize these neurons at frequencies sufficient to cause depolarization blockade and homeostatic inhibition of other midstream inputs. Therefore stimulation may behave as lesions in both the stimulation target and the downstream area.

Figure 1D shows the predicted effect of stimulation above 200 Hz. This frequency may be above the maximum firing rate of the stimulation target neurons, and depending on stimulation parameters could maintain these neurons (and nearby axons and dendrites) in a chronically depolarized state. In this case the neurons will not fire, and there will be no output from the target region, either due to the DBS or due to the upstream activity. This releases downstream neurons from depolarization block so that the midstream signals can once again propagate to the output.

We emphasize that this model, the existence of the different neuron populations, and the specific frequencies at which these effects occur are conjectures based upon known properties of single synapses and neurons rather than experimentally-verified network properties. It is intended to illustrate potential phenomena that are in need of further investigation. In different brain regions, disease states, or behavioral conditions some, all, or none of these phenomena may be relevant. The model does not take into account the membrane properties of particular neurons, the presence of inhibitory neurons, the effect of inhibition or excitation on rebound firing, and the presence of recurrent and reciprocal inputs. As detailed measurements in specific disease states become available, it is hoped that these measurements can be incorporated into and constrain DBS models to provide an increasingly faithful prediction of the response to different stimulation parameters.

## Childhood secondary dystonia

There is very limited understanding of the interaction between DBS and childhood acquired dystonia. Based on the clinical experience at our center and others, stimulation in GPi can reduce the hypertonic component of dystonia in 50–70% of children, depending on severity, diagnosis, and other associated impairments (spasticity, dyspraxia, contractures, etc.). Although early attempts at functional neurosurgery in children reported significant reduction in dystonia from lesions in thalamus (Cooper et al., 1980, 1982), only recently have we and other centers started to consider the thalamus as a target for DBS (Vercueil et al., 2001; Gruber et al., 2010). Preliminary anecdotal observations (unpublished) suggest that stimulation in Voa/Vop or Vim nuclei of the thalamus is capable of reducing the hyperkinetic components of dystonia in a subset of children. Children with both hypertonic and hyperkinetic impairments may benefit from a combination of stimulation in GPi and motor thalamus (unpublished).

Standard procedures for DBS targeting often include the use of microelectrode recording (MER) in the operating room to confirm the location of the electrode in GPi, as well as test stimulation to assess for side effects (Air et al., 2011; Olaya et al., 2013). However, MER and stimulation require children to be awake during surgery, and this often cannot be achieved safely due to respiratory risks, behavior, or concerns for dystonic spasms while in a stereotaxic head frame.

More recently, we have developed a procedure based on chronic recording for determination of epileptic foci. The “Stereo-EEG” procedure used as part of phase 2 epilepsy resection surgical planning involves implantation of temporary recording and stimulating electrodes at multiple potential surgical sites. We use the same technology to implant temporary recording and stimulating electrodes at potential sites for permanent electrode placement. The standard surgical procedures are well-described elsewhere, but the significance for purposes here is that we have the ability to record simultaneously from multiple depth electrodes placed in bilateral GPi and motor thalamus. Recording and test stimulation are subsequently performed with the child awake in the epilepsy-monitoring unit. This procedure is not only safer, but it yields much more relevant targeting information since recording and testing can be performed with the child unrestrained. Once the optimal targets have been identified, the temporary electrodes are removed and permanent electrodes placed under general anesthesia. The significance for the model proposed here is that the procedure provides long-term simultaneous recording from multiple locations in GPi and thalamus.

### Methods—deep brain recording

In contrast to standard MER electrodes that have typical impedance of 1 MOhm or more, the impedance of the stereo-EEG depth electrodes is only 70–90 kOhm with a significantly larger contact surface area. Therefore the stereo-EEG electrodes are expected to have higher levels of background noise and lower sensitivity to the presence of low-amplitude spikes. We expect that the stereo-EEG electrodes will underestimate both the number of active cells and their average firing rates, although the relative firing rate changes due to movement or behavioral state changes will be preserved.

### Results—deep brain recording

The average firing rate recorded on MER during surgery in GPi of children with acquired dystonia (5–10 Hz) (McClelland et al., 2016) is typically lower than reported for genetic (primary) dystonia, and much less than reported in Parkinson's disease and healthy non-human primates (Zhuang et al., 2004; McClelland et al., 2016). However, the distribution of firing rates in other disorders is broad (for example, relatively slowly-firing cells occur in Tourette Syndrome) so this may represent relative preservation of the slowly-firing cells rather than a decrease in firing rate of faster-firing cells. It is not unusual during surgery to find an MER pass through GPi that is completely silent (McClelland et al., 2016). Despite this, implantation of permanent stimulation electrodes even within silent regions of GPi can be effective (unpublished observation).

The average firing rate recorded from stereo-EEG electrodes in GPi is typically 1–5 Hz during activity and drops to zero at rest or when asleep. This is likely an underestimate of the rates recorded using MER, since GPi is known to fire at much higher rates at rest and with dystonic spasms (Zhuang et al., 2004). Despite this, the important observation from simultaneous electrode recordings is the widespread activation in both GPi and motor thalamus, and the observation that both GPi and thalamus increase the firing rate during movement. This is not the prediction of the rate model, in which the inhibitory output of GPi would be expected to correlate inversely with motor thalamus. Many authors have addressed this apparent paradox (Goldberg et al., 2013), and possibilities include a separate movement-related driving signal to thalamus (most likely from cortex) or rebound excitation of thalamus (Zhuang et al., 2004; Rovo et al., 2012).

**Figure 2:**
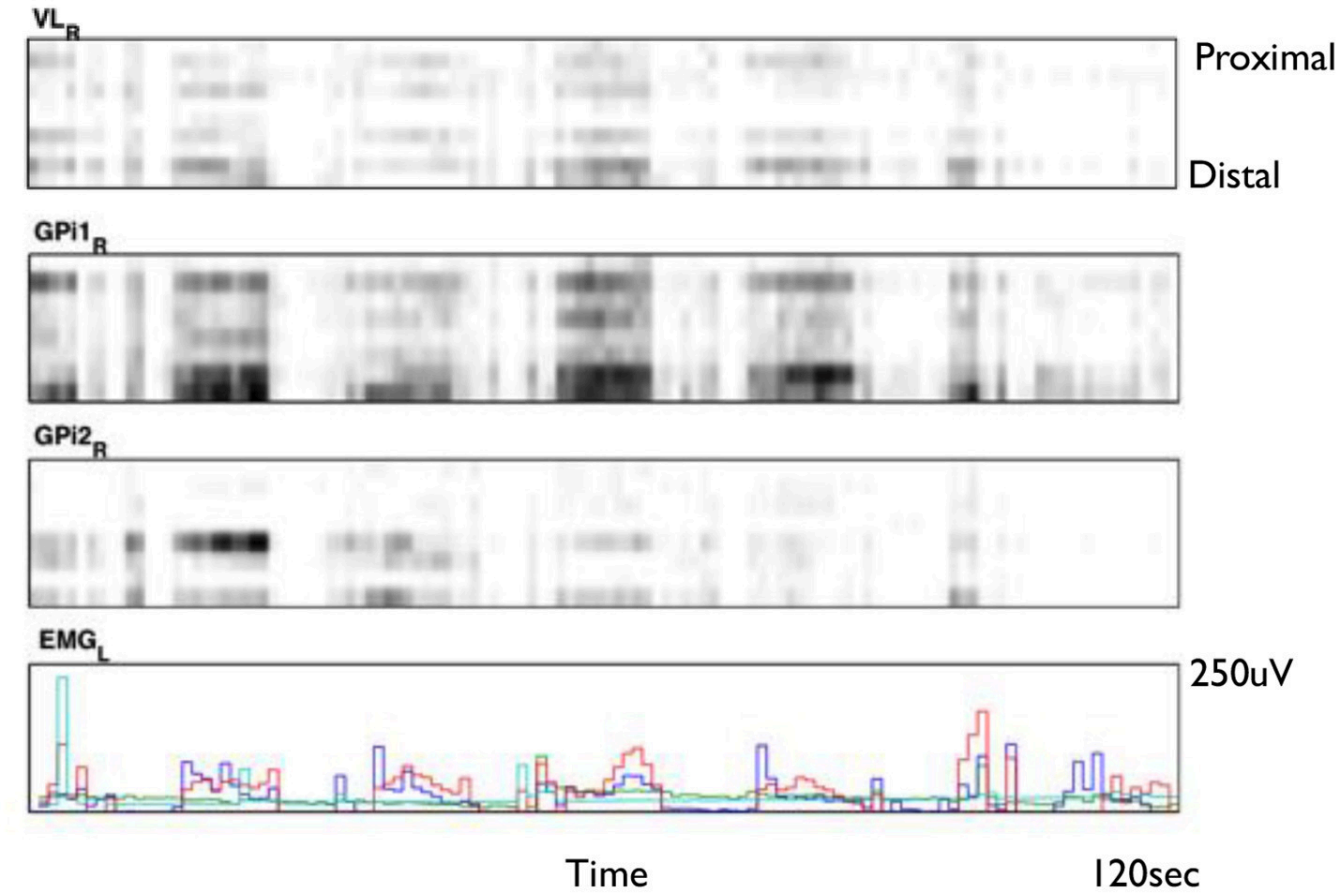
Smoothed spike rasters from right ventrolateral nucleus of the thalamus (VL) and two different 8-contact electrodes in right globus pallidus internus (GPi) during passive extension and flexion of the left knee in a child with static hemidystonia. Each raster shows eight different contacts from proximal **(top)** to distal **(bottom)** recorded over a 2 min period. Gray level is proportional to the total number of spikes in each 100 ms bin, normalized for each brain region: black represents the largest number of spikes seen in each region during 100 ms, while white corresponds to no spikes. Bottom trace shows EMG from left biceps (red), triceps (blue), flexor carpi radialis (aqua), and extensor carpi radialis (green) (Clinical data, previously unpublished).

**Figure 3:**
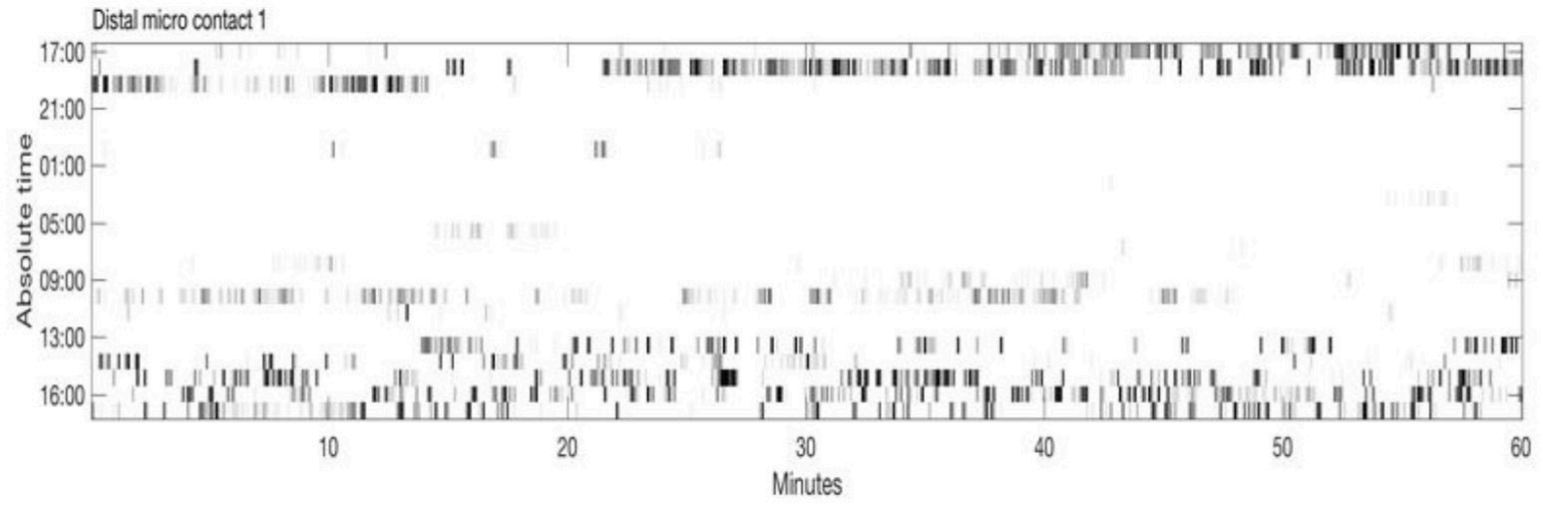
Twenty-four hour extracellular microelectrode recording from a single neuron in Voa/Vop nucleus of the thalamus. Each row of the raster shows 1 h of recording. Hours from 17:00 until 16:00 the following day are indicated at the left. Gray level is proportional to the total number of spikes in each 20 s bin: black represents the largest number of spikes seen during 20 s, while white corresponds to no spikes (Clinical data, previously unpublished).

Figure 2 shows an example of spike rasters from stereo-EEG. Average firing rates vary with activity and diagnosis. Typical spike rates recorded with these electrodes are 1–5 Hz during movement and 0–1 Hz while relaxed and awake. It can be seen that activity in both GPi and ventrolateral thalamus (VL) correlate with the EMG and do not anticorrelate with each other. Figure 3 shows 24 h of recording from a single neuron in motor thalamus. Average firing rate for this cell was 500 spikes per hour.

The importance of these results for our DBS models is to understand that a relatively low firing rate in GPi or thalamus does not preclude the effectiveness of stimulation. As shown in Figure 1, the effect of stimulation may be to suppress input to the upstream cells, perhaps in GPi, while simultaneously limiting the transmission and output from downstream. If destruction of cells in GPi leads to homeostatic plastic changes in thalamus, then DBS might not only block propagation of abnormal signals from GPi, but it might also restore normal thresholds for other thalamic inputs. We thus hypothesize a combined effect that is different from a lesion, because of the potential to normalize downstream sensitivity.

### Methods—simulation

Almost all of the model components described above have been developed or simulated previously. The significance of the synthesis here is to apply this to childhood secondary dystonia and to show how various mechanisms of DBS activity may work on populations of neurons within different parts of the network responsible for dystonia. In order to provide a further demonstration of the combination of some of ideas above, we perform simple simulations of mechanisms by which DBS can interfere with abnormal signals in basal ganglia and thalamus.

### Simulation of deep brain stimulation

To simplify calculation and ensure robust selection of a small number of neural parameters, we use the Izhikevich neuron model (Izhikevich, 2003) which provides an approximation to the time-varying membrane potential predicted by the Hodgkin-Huxley equations. We do not simulate synaptic or homeostatic plasticity, nor do we simulate inhibitory output neurons. Therefore, this simulation is most applicable to DBS applied to thalamus, with upstream inputs from cortex (depolarizing) and GPi (hyperpolarizing) and downstream excitatory output to cortex.

Four populations of cells are simulated. Four hundred thalamic neurons with Izhikevich parameters for “thalamocortical neurons” with excitatory projections to cortex, 400 cortical neurons with “regular spiking” Izhikevich parameters and with excitatory recurrent connections to cortex and output connections to primary motor areas, 100 cortical neurons with “fast spiking” Izhikevich parameters and inhibitory recurrent connections throughout cortex as well as output connections to primary motor areas, and 400 neurons with “regular spiking” parameters in primary motor areas that will ultimately be responsible for movement. This is a highly oversimplified model, but it serves to illustrate the concepts proposed here.

All areas have random recurrent connections to allow for sustained activation. Thalamocortical cells project 1:1 onto the excitatory cortical population. Excitatory cortical cells project 1:1 onto output cells. Inhibitory cortical cells project diffusely onto output cells. Connection strengths are set empirically to permit excitability yet prevent sustained oscillation. DBS is applied to all thalamic cells with a pulsewidth of 180 us, amplitude of 1.5 mA (current mode), and varying frequencies between 5 and 250 Hz.

### Simulation results

**Figure 4:**
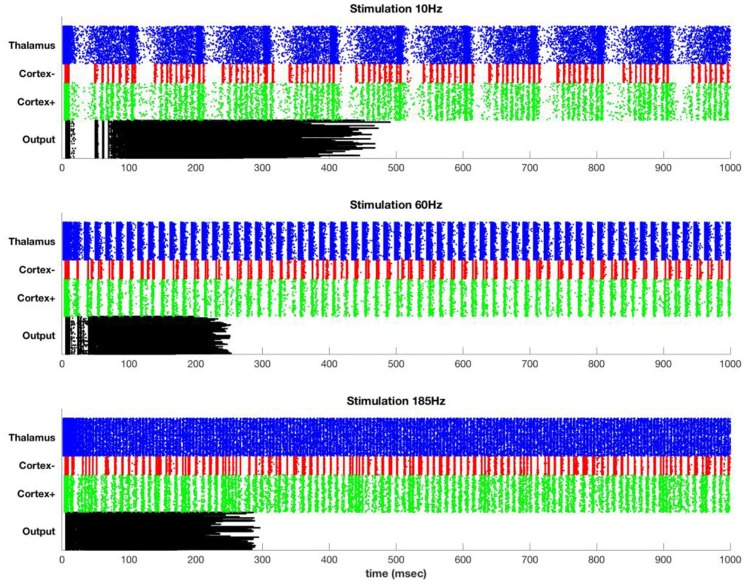
Stimulation at 10 Hz **(top)**, 60 Hz **(middle)**, and 185 Hz **(bottom)** in the simulated model. Each row represents a single simulated neuron [400 thalamus (blue), 100 inhibitory cortical (red), 400 excitatory cortical (green), and 400 motor output (black)]. Thalamus has sufficient intrinsic drive that it fires at high rates except for during the refractory period following a DBS pulse. The DBS frequency persists in cortex but is attenuated at the output.

Figure 4 shows typical results for stimulation at 10, 60, and 185 Hz. At 10 Hz, between DBS pulses there is time for recurrence of background thalamic activity, and this activity transmits to the cortex. However, as the DBS frequency increases, more and more of the thalamic activity is due to the DBS pulses themselves, which effectively substitute for and block the endogenous activity. For example, although cortex shows high activation at 185 Hz, almost all of this activation is due to the DBS pulses themselves.

The DBS component of the cortical activity is attenuated at the output due to the effect of the inhibitory interneurons. In particular, widespread synchronous activation of cortex causes a coordinated volley of inhibition that affects both the cortex and the cortical output. After an initial build-up period, this inhibition is so strong as to block all further output. This is only one mechanism by which repetitive or excessively synchronous signals might be attenuated. Lateral inhibition is another possibility, and special circuitry may be present to prevent or terminate seizure-like activity. The simulation here is intended to illustrate that even in the absence of special circuitry, attenuation of overpowering synchronous signals could occur due to the normal action of inhibitory interneurons.

**Figure 5:**
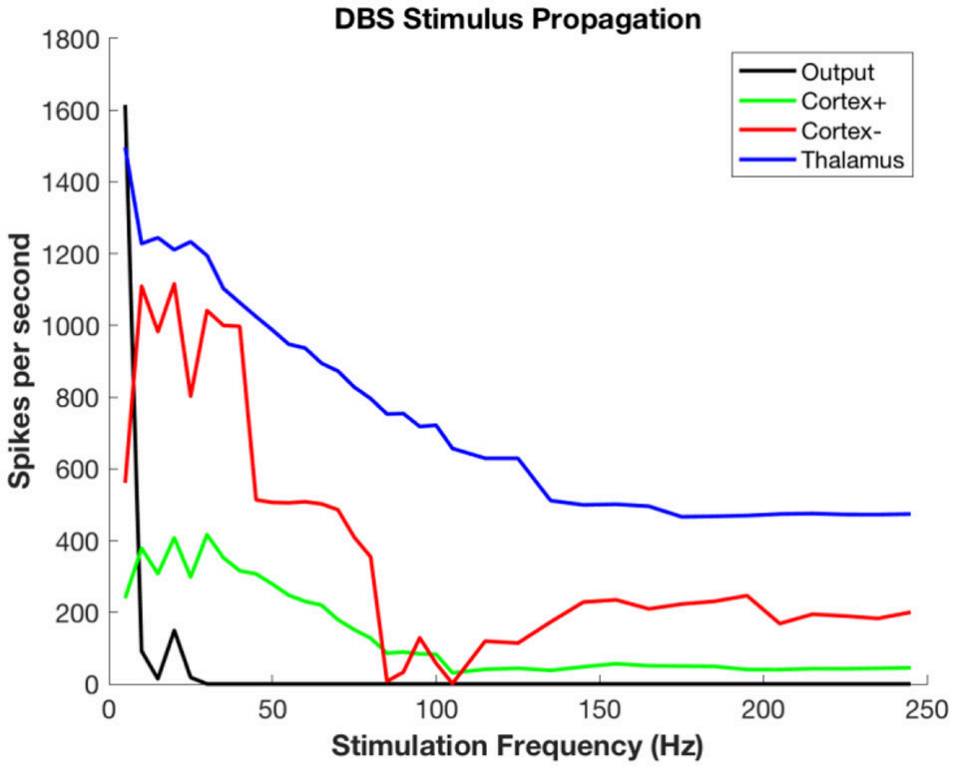
Propagation of stimulus-related activity per stimulus pulse as a function of DBS stimulation frequency.

Figure 5 shows the propagation of the DBS pulses through the system. The spike count attributable to the DBS pulses decreases even in the target (in this case, thalamus) because there is less time between pulses for rebound activity. Propagation to both excitatory and inhibitory neurons of cortex has the same phenomenon, with decreases due to refractory periods in cortex and decreasing ISI for rebound activity. The output is zero for all but the slowest rates due to the widespread synchronous activity causing massive generalized inhibition.

**Figure 6:**
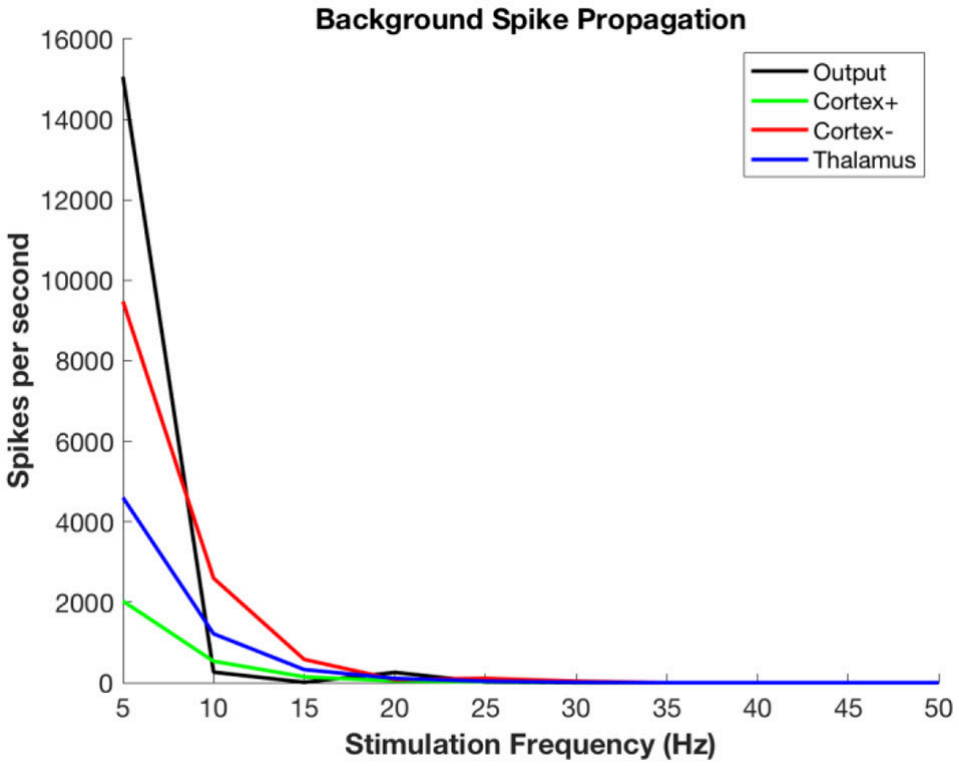
Propagation of non-stimulus-related background firing in thalamus as a function of DBS stimulation frequency.

Figure 6 shows the propagation of the background thalamic activity to other regions. Note that beyond stimulation frequencies of ~20 Hz there is no propagation at all. This is because the DBS pulses overwhelm the background and then no further activity is possible during the refractory period. This happens in all cell populations. The actual frequency at which propagation ceases will depend upon details of the absolute and relative refractory periods, the relative strength of stimulation, local cell interactions, and other factors that are not included in this model. The purpose here is to illustrate the substitution of DBS-related activity for the background signal-related activity.

The figures are intended to reflect stimulation in thalamus, for which much of the clinical effect is immediate. In order to model stimulation in GPi, it would be necessary to include not only plasticity effects, but also to be consistent with the inhibitory output from GPi. This would require another source of excitatory drive to thalamus, which is most likely to be cortical in origin (Goldberg et al., 2013). Development of such a model would be fascinating and potentially tremendously useful for clinical prediction, but it is beyond the scope of the much simpler hypotheses advanced here.

## Discussion

These results show that a very simple model of pallidal, thalamic, and cortical population interaction explains how DBS can block involuntary movement while not causing significant motor side effects. The primary mechanism is conversion of an effective but involuntary pattern into an ineffective pattern. The spatial homogeneity and temporal rhythmicity of the stimulation are ineffective patterns that can interfere with propagation of dystonic signals while not themselves causing dystonia or other involuntary muscle contractions. Ineffectiveness of spatially homogeneous rhythmic patterns may be due to normal mechanisms that are sensitive to and thus propagate spatial and temporal variability. It could also be due to mechanisms that are designed to detect and inhibit seizure-like activity.

Inhibition of involuntary activity may operate through different mechanisms depending on the frequency and location of stimulation. Since response in GPi is not immediate, the effect of GPi stimulation is likely mediated in part by synaptic plasticity mechanisms at downstream synapses, either pallido-thalamic or thalamocortical synapses. At typical stimulation frequencies, long-term potentiation (LTP) would be expected, but long-term depression (LTD) could potentially be induced at frequencies below 1 Hz. Plastic effects might potentiate the inhibition of thalamus, or might enhance the transmission of the DBS signal to downstream synapses. It is unlikely that plasticity mediates the entire effect however, since even following chronic GPi stimulation there is immediate return of dystonic symptoms when batteries fail, and immediate return of therapeutic effect when batteries are replaced. Therefore, it is likely that the effect of GPi stimulation is mediated by a combination of long-term plasticity and immediate stimulation effects.

The role of homeostatic plasticity mechanisms in the adaptation to DBS has not previously been investigated. We do not know whether or not they are relevant, but the widespread presence of this phenomenon suggests that it could be a potential contributor. If so, then an important effect of chronic DBS could be alterations in firing threshold of downstream neural populations. DBS would therefore behave as a “remote lesion”, with inhibitory effects both at the site of DBS (due to depolarization blockade and substitution of rhythmic for background pattern) and at the downstream synapses (due to the previous effects plus homeostatic changes in firing threshold). Homeostatic changes would be expected to propagate throughout the basal ganglia and cortical network, wherever the DBS signal is observable. Thus DBS is a “network” intervention and may have widespread neuromodulatory effects (McIntyre et al., 2004b; Wichmann and DeLong, 2016). Furthermore, the lack of DBS propagation to primary motor output areas suggests that the neuromodulation has little or no effect on plasticity in spinal cord.

Different stimulation frequencies may have very different effects, and this fact may permit tuning of therapy to the particular needs of individual children. In particular, some component of the normal voluntary (and involuntary) activity in pallidum may be allowed through if stimulation frequency is below the maximum entrainable frequency of GPi or thalamus. At higher frequencies, all endogenous signals will be blocked and ineffective stimulation substituted. At even higher frequencies there may be no transmission of the stimulation at all as neurons remain depolarized and cease firing.

While the model proposed here is consistent with clinical observations, there remain several inconsistencies with recording data. Our data suggest that firing rates in thalamus and GPi are very low at baseline and increase to only a few spikes per second during activity. This is a much lower firing rate than seen in healthy nonhuman primates and in human subjects undergoing intraoperative MER for dystonia and other movement disorders (Zhuang et al., 2004). Due to our recording equipment, our rate estimates are below those seen with MER in other children with secondary dystonia (McClelland et al., 2016), although it remains likely that low firing rates are a characteristic feature of secondary or acquired dystonias. This suggests that inhibition of abnormal activity may not be the primary mechanism of efficacy. On the other hand, chronic low activity could lead to upregulation of downstream receptors, so that if thresholds are very low then it would become important to block even low levels of incoming activity. The output of GPi is inhibitory, yet both GPi and its thalamic targets increase activity in response to movement. This suggests that the effect of GPi on thalamus is to “sculpt” ongoing activity rather than as a general rate inhibition. Understanding these phenomena will require considerably more recorded data from a larger variety of children and adults.

As more data from human electrophysiology become available it will be increasingly important to have more accurate models of DBS functionality that predict target location and optimal stimulation parameters. It seems clear that the effect of DBS is more complex than simply an increase or decrease in firing rate. Understanding the relationship between DBS and the normal and abnormal propagation of signals in basal ganglia and thalamus will allow a path toward improvement in therapy and more accurate selection of patients, with the ultimate goal of preventing the clinical manifestations of dystonia.

## Author contributions

TS is responsible for the theory, simulations, clinical reports, patient selection, patient programming, outcomes observations, data quality assessment, data interpretation.

### Conflict of interest statement

The author declares that the research was conducted in the absence of any commercial or financial relationships that could be construed as a potential conflict of interest.
